# Planetary Boundary for Novel Entities: Time for a Reboot

**DOI:** 10.1021/acs.est.6c03044

**Published:** 2026-07-02

**Authors:** Annika Jahnke, Yuge Bai, Werner Brack, Bethanie Carney Almroth, Miriam L. Diamond, Anna Lia S. Tromer Dragsdahl, Beate I. Escher, Peter Fantke, Dana Kühnel, Matthew MacLeod, Ad M. J. Ragas, Boris Sakschewski, Christian Schmidt, Volker Strauss, Gabriele Treu, Patricia Villarrubia-Gómez, Zhanyun Wang, Katrin Wendt-Potthoff, Marlene Ågerstrand, Levke Caesar

**Affiliations:** † Helmholtz-Centre for Environmental Research - UFZ, Permoserstr. 15, 04318 Leipzig, Germany; ‡ RWTH Aachen University, Worringerweg 1, 52074 Aachen, Germany; § Potsdam Institute for Climate Impact Research (PIK), Member of the Leibniz Association, P.O. Box 6012 03, D-14412 Potsdam, Germany; ∥ Institute of Ecology, Evolution and Diversity, Goethe University, Max-von-Laue-Straße 13, 60438 Frankfurt am Main, Germany; ⊥ Department of Biological and Environmental Sciences, University of Gothenburg, Box 463, 405 30, Göteborg, Sweden; # Department of Earth Sciences, 7938 University of Toronto, Toronto, Ontario M5S 3B1, Canada; 7 School of the Environment, University of Toronto, Toronto, Ontario M5S 3E8, Canada; 8 Section of Quantitative Sustainability Assessment, Department of Environmental and Resource Engineering, Technical University of Denmark (DTU), Bygningstorvet, Building 115, 2800 Kgs. Lyngby, Denmark; 9 Centre for Absolute Sustainability Assessment, Technical University of Denmark (DTU), 2800 Kgs. Lyngby, Denmark; 10 Environmental Toxicology, Department of Geosciences, Eberhard Karls University Tübingen, Schnarrenbergstr. 94-96, 72076 Tübingen, Germany; 11 substitute ApS, Graaspurvevej 55, 2400 Copenhagen, Denmark; 12 Department for Evolutionary Ecology and Environmental Toxicology, Goethe University, 60438 Frankfurt Am Main, Germany; 13 Department of Environmental Sciences, College of Agriculture and Environmental Sciences, University of South Africa, Florida, 1710 Roodepoort, South Africa; 14 Centre for Mineral Biogeochemistry, University of the Free State, Bloemfontein, Free State 9301, South Africa; 15 Department of Environmental Science, 7675 Stockholm University, Svante Arrhenius väg 8, 114 18 Stockholm, Sweden; 16 Radboud Institute for Biological and Environmental Sciences (RIBES), 6029 Radboud University, Heyendaalseweg 135, 6525 AJ Nijmegen, The Netherlands; 17 German Environment Agency (UBA), Wörlitzer Platz 1, 06844 Dessau, Germany; 18 Department of Wildlife, Fish, and Environmental Studies, Swedish University of Agricultural Sciences, Skogsmarksgränd 17, 901 83 Umeå, Sweden; 19 Stockholm Resilience Centre, Stockholm University, 106 91 Stockholm, Sweden; 20 Empa−Swiss Federal Laboratories for Materials Science and Technology, Lerchenfeldstrasse 5, 9014 St. Gallen, Switzerland; 21 National Centre of Competence in Research (NCCR) Catalysis, 8093 Zürich, Switzerland; 22 Helmholtz-Centre for Environmental Research − UFZ, Brückstr. 3a, 39114 Magdeburg, Germany; 23 University of Southern Denmark, Department of Green Technology & Danish Institute for Advanced Study (DIAS), 5230 Odense, Denmark

**Keywords:** chemicals and materials, Earth system stability, risk management, pressures − states − impacts control variables, quantitative indicators, global (environmental) monitoring, environmental governance, absolute environmental sustainability assessment, safe operating space

## Abstract

Novel entities are diverse and complex; hence, defining their Planetary Boundary through control variables, indicators, and acceptable limits is challenging. Here, we highlight shortcomings of the current quantitative boundary, which applies a purely precautionary interpretation of safety that defines the safe operating space as zero novel entities that are not fully characterized. In practice, the zero limit is unattainable and undesirable for human societies that rely on services provided by novel entities. Furthermore, it is not operational within the framework of other boundaries, which have risk-based thresholds. We therefore propose a reboot of the Planetary Boundary for Novel Entities and recommend governance and monitoring strategies based on: (a) clarification of the “safe operating space” for novel entities, distinguishing pure precaution (“safe from the unknown”) from operational precaution that tolerates a workable, non-zero level of uncertainty; (b) complementary, multilayer control variables spanning pressures, states, and impacts; (c) extensions to existing monitoring systems to provide quantitative indicators of distribution, accumulation, exposure, and impacts for subsets of novel entities to communicate the status and trends of stress from novel entities on the Earth system; and (d) recognition of regulatory and policy-related success stories that illustrate comprehensive, protective, and operational governance of novel entities.

## Graphical abstract

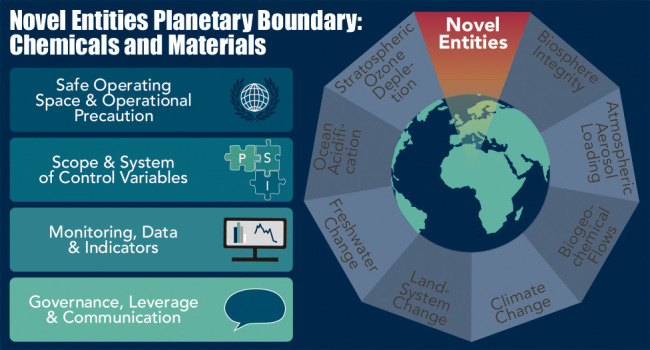

## Background and Scope

The Planetary Boundary framework characterizes human-induced pressures that lead to Earth system destabilization and increased risk of reducing its habitability. Within the framework, the Planetary Boundary for Novel Entities comprises a vast diversity of anthropogenic and naturally occurring chemicals and materials, newly created or mobilized at scales capable of perturbing Earth system processes, characterized by persistence and mobility, often combined with poorly understood impacts. They exert pressure along technology life cycles (resource extraction, production, use, and end-of-life treatment), leading to impacts via fate and distribution, accumulation and exposure, and negative effects.^1–6^ Novel entities can destabilize the Earth system in many ways, including impacts on biodiversity, climate (e.g., through changes in albedo), habitats, regenerative food supply, and biogeochemical cycling.^6–10^ Novel entities may be produced and globally distributed long before they are detected and their impacts are understood, attributed, or assessed. Substantial data gaps arising from confidential business information (e.g., data relevant for safety assessment),^11^ the presence of complex substances of unknown or variable composition, uncharacterized transformation products, limited safety assessment, and very restricted global monitoring coverage, make their assessment and management highly challenging.^12,13^

### Why the Planetary Boundary for Novel Entities Needs a Reboot

After more than a decade of remaining “not yet defined”, Persson et al.^3^ introduced a qualitative, weight-of-evidence approach to the Novel Entities boundary. Based on past experience with large-scale pollution issues, they argued that the boundary was transgressed because the pace of introduction, production, and releases of novel entities exceeded the capacity for safety assessments, monitoring, and management. Richardson et al.^5^ later translated these arguments into a quantitative boundary for the safe operating space for novel entities of zero, asserting that “the only truly safe operating space [ . . .] is one where these entities are absent”. The current boundary is logical and consistent with a stringent definition of precaution.^14^ However, it is inconsistent with the other Planetary Boundaries, which define safe operating spaces through risk-based thresholds aligned with the “working definition” of precaution (Box [Boxed-text box1-fo]).^14^ Furthermore, this definition is impractical because of societal reliance on novel entities, and hence their continued production, and insufficient capacity to conduct comprehensive risk assessments for the growing number and diversity of novel entities. As a result, the current boundary is not operational since it does not provide indicators to assess the severity, distribution, or evolution of planetary-scale risks from a widely varying range of novel entities, nor does it support prioritization among compound groups, impacts, or affected regions.^15^

1 **Box 1:**

**Defining the safe operating space**. The goal of the Planetary Boundary framework is to define a “safe operating space for humanity” as a set of risk-based thresholds. For novel entities, uncertainty and ignorance of impacts are intrinsic: most novel entities are poorly characterized, impacts may only manifest with long delays and far from sources, and those impacts may be practically irreversible.^3^ Hence, some might interpret “safe” as “safe from the unknown”, which logically leads to a **purely precautionary boundary** of zero.^5^ However, such a boundary is non-operational since societal benefits of novel entities are ignored. Here, we interpret the “safe operating space” for novel entities in a way that is consistent with the rest of the Planetary Boundary framework, i.e., as a **zone of operational precaution under uncertainty**. This framing acknowledges that zero release of novel entities is practically unattainable and uncertainty remains central, while emphasizing the need to prevent the combination of stressorsfrom exceeding levels that could erode Earth system resilience.

### Scope of This Paper

We describe four conceptual building blocks for the Planetary Boundary for Novel Entities to be workable within the Planetary Boundaries framework: (a) the interpretation of a “safe operating space” for novel entities and its implications for operationalizing precaution (Box [Boxed-text box1-fo]); (b) clarifying the scope of novel entities and proposing a system of complementary control variables spanning pressure, state, and impact (Section 1.2.1), (c) improving data coverage and availability to provide quantitative indicators for the control variables (Section 1.2.2); and (d) highlighting regulatory and policy-related success stories to identify leverage points for reducing planetary risk from novel entities and strategies for improved communication (Section 1.2.3).

## 1 Control Variables and Interactions of Planetary Boundaries

### Scope of the Novel Entities Boundary

To maintain conceptual clarity and minimize overlap with other Planetary Boundaries, a clear definition of “novel entities” is required. In the context of the Planetary Boundaries framework, novel entities comprise anthropogenic and naturally occurring chemicals, materials, and other human-generated entities that have the potential to disrupt Earth system processes through biological or physical effects, whose likelihood and scale of impact are increased by persistence, mobility, accumulation, and delayed detectability. In the present Perspective, we focus primarily on chemicals and materials as the most tractable components of the Novel Entities boundary and therefore suggest using the subtitle “Chemicals and Materials” for more clarity. We further propose that engineered biological systems (e.g., genetically modified organisms) should be considered outside the definition of novel entities and, instead, should be considered under the Biosphere Integrity boundary, given their primary modes of action via ecological interactions, replication, and gene flow. Anthropogenically mobilized elements (including metals, rare earth elements and radionuclides) could, by analogy, be considered under the Biogeochemical Flows boundary. However, since they can be assessed using a system of control variables representing their impact pathway,^16^ we consider them within the scope of the Novel Entities boundary.

### Requirements for Control Variables

Novel entities in the environment lead to multifaceted impacts that are, in practice, impossible to fully characterize. Their complexity requires defining a broad set of control variables and related indicators (Box [Boxed-text box2-fo]). Three criteria apply for control variables:^3^ they must be Earth-system-relevant, reflect evidence of destabilizing behavior or eroding Earth-system resilience, and be observable by means of quantitative indicators, preferably at a cross-regional or global scale. Applying these criteria to novel entities poses considerable challenges related to (lack of) knowledge, observability, and causality for the enormous number of novel entities. We conclude that operationalizing the Novel Entities boundary requires a flexible, integrated system of control variables that accounts for uncertainty, delayed effects, and systemic interactions. The Novel Entities boundary should enable accounting for boundary interactions, as pressures from novel entities can propagate across and affect multiple Earth system processes including climate and biosphere integrity.

2 **Box 2:**

**Control variables vs. indicators**. Within the Planetary Boundary framework, **control variables** are planetary-scale parameters that regulate the state and functioning of a Planetary Boundary process or system and are traceable to anthropogenic stressors to assess human influence on Earth system stability. For most boundaries, this process is clearly defined (e.g., radiative forcing for climate change). For novel entities, however, no single Earth system process can be identified, as they affect multiple Earth system elements and boundaries. These interactions complicate the identification of appropriate control variables. **Indicators** are measurable quantities, causally linked to control variables, that enable their operationalization (i.e., their practical assessment, monitoring, and comparison across time and space). Control variables and indicators must be “fit-for-purpose” according to the boundary. For some boundaries, control variables are directly observable and therefore used as indicators, such as *atmospheric carbon dioxide concentration* for the Climate Change boundary. In other cases, control variables are conceptual and require representation by one or more observable indicators. For example, for the Biosphere Integrity boundary, the control variable *genetic diversity* is operationalized using the indicator *extinctions per million species-years*.

### Three Control Variables for Novel Entities Representing Pressure, State, and Impact

We propose that the Novel Entities boundary should be understood as a dynamic container for chemical and material stressors whose planetary relevance may be well established, emerging, or not yet recognized. Given the sheer number of chemicals and materials, a substantial fraction remains uncharacterized.^3,5^ Following a generalized impact pathway (life cycle emissions, distribution, accumulation, exposure → impacts),^17^ at least three control variable types should be included covering pressure (extraction, production, waste generation), state (use, distribution, accumulation, exposure), and impact. Individually, each type captures only part of the risk posed by chemicals and materials, but together, they complement each other regarding the level of prevention (versus reaction), proximity to Earth system impact, and management/governance feasibility (Figure 1). The proposed pressure, state, and impact control variable types describe (i) extraction and production pressures, (ii) environmental distribution, accumulation, and exposure, and (iii) Earth system impacts of chemicals and materials. They are not directly measurable when addressing the high number and diversity of chemicals and materials. Therefore, they must be operationalized with indicators that could be globally quantifiable using standardized methodologies or harmonized modeling, and with sustained international efforts to establish, strengthen, and harmonize open and interoperable data systems.

**Figure 1:**
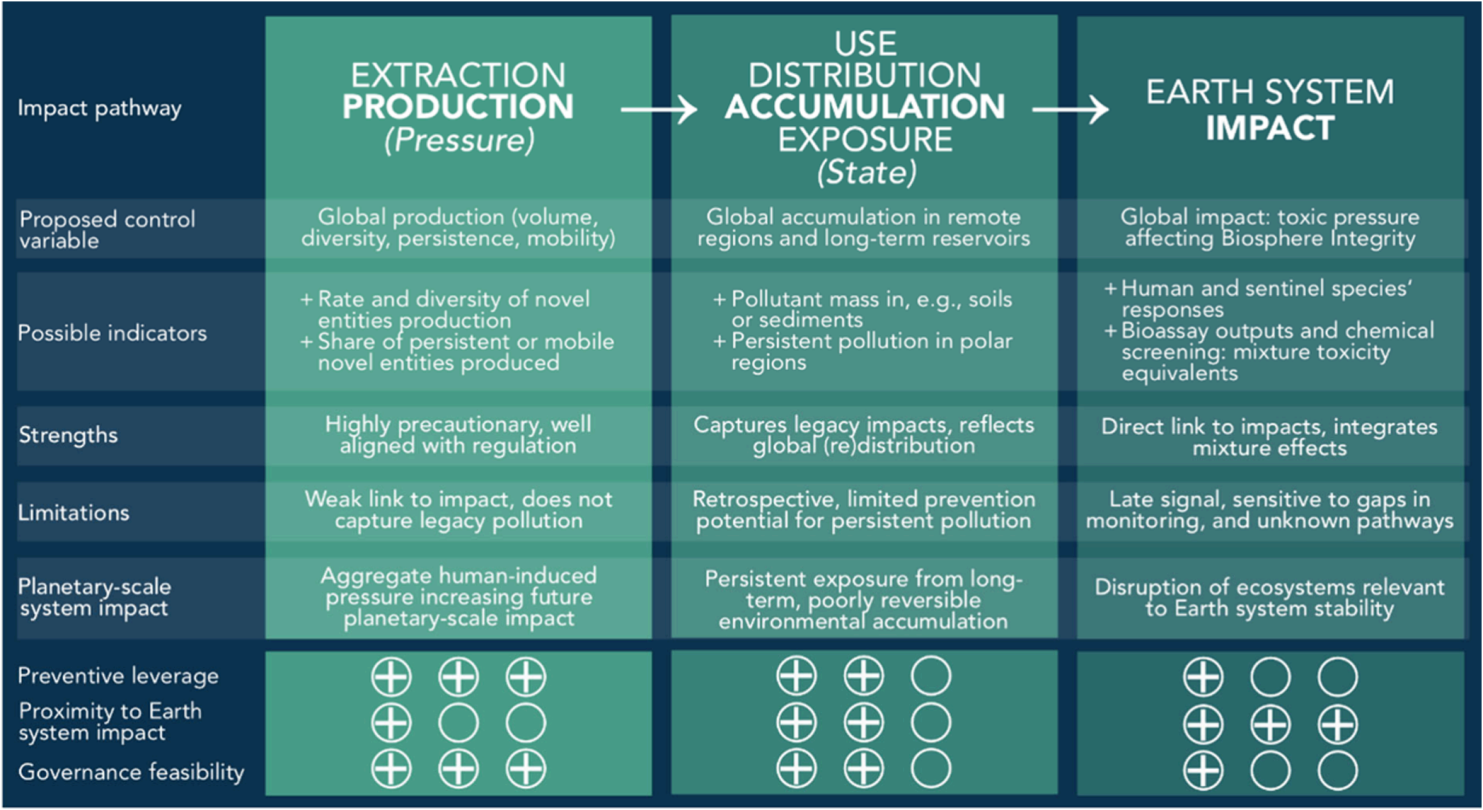
Conceptual framework for operationalizing the Novel Entities boundary using complementary control variables covering pressure, state, and impact, positioned along the extraction, production → use, distribution, accumulation, exposure → impact pathway. The bottom panel provides a qualitative comparison with respect to preventive leverage, proximity to Earth system impacts, and management/governance feasibility. Circles with “+” symbols indicate higher relevance or performance relative to the other types. Complementary use of the three control variable types ensures robust assessment.

### a Production (Pressure) Control Variable

The “production” control variable represents chemical and material proliferation early in the impact pathway, with the potential to support preventive management. In addition to intentional production, anthropogenic release pathways linked to unintentional generation such as byproduction, inappropriate waste management, including open burning of agricultural biomass residues, municipal solid waste, and plastic debris, may contribute substantial emissions and environmental exposure. Quantitative indicators for the “production” control variable could include rates of resource extraction, production volumes, waste generation, and their change over time, measures of chemical and material diversity, and share of produced novel entities with high potential to cause poorly reversible planetary impacts due to high persistence or mobility in the environment. Chemicals and materials can also continue to be present due to the “lock-in” of production and use and/or the accumulated stock of a chemical or material from which releases are slow.^18,19^ A key challenge is that relevant data are incomplete across novel entities and regions, dispersed across jurisdictions, and in many cases subject to confidentiality constraints.^11,14,17,20^ Selected indicators of production and waste generation should align with existing or emerging regulatory or policy frameworks to maximize reporting efficiency. However, data availability, accessibility, and transparency vary strongly across regions: in many countries, record keeping and monitoring infrastructures remain incomplete, difficult to access, or absent (see Section 1.2.3). These limitations constrain the choice, quality, and comparability of indicators to operationalize this control variable.

### b Accumulation (State) Control Variable

The “accumulation” control variable represents the distribution, accumulation, and exposure to novel entities, in particular of wildlife because of close links with the Biosphere Integrity boundary, and in remote regions as indicators of poorly reversible planetary pollution. Quantitative indicators could include concentrations in biodiverse regions (e.g., Amazonia) where related ecotoxicological impacts may be particularly consequential, remote areas (e.g., polar regions, high-altitude mountains) as indicators of long-range transport and persistence, and in environmental media that act as long-term reservoirs for pollution, such as sediments and areas for which recovery is very slow (e.g., deep seas) or impacts are very high. Analytical tools are being developed to capture ever more novel entities. Furthermore, modeling is advancing to provide more robust and comprehensive predictions (depending on data availability). Indicators of accumulation should capture past and present emissions, cover environmental stocks of both legacy and emerging novel entities, and identify cases where emissions and exposure remain high even for nonpersistent substances. They should be adaptable to changes in analytical capabilities and capacities, changes in emissions, market pressures, and regulatory interventions.

### c Impact Control Variable

The “impact” control variable represents the extent to which novel entities affect ecosystem resilience. We propose to consider mixture effects in cell-based bioassays as a promising indicator, to be tested and validated in recurring applications, since current substance-specific regulatory approaches systematically underestimate impacts from complex mixtures.^20,21^ Examples include mixture toxicity equivalents in water, soil, or biota,^22^ derived using new approach methodologies (NAMs)^23^ that determine toxicity of chemical mixtures from environmental media comprising known and unknown compounds, including transformation products. We propose mixture effects as additional indicator, predicted from measured or estimated concentrations, such as toxic units, potentially affected or field-observed disappeared fractions of species.^24–27^ Such indicators integrate the combined effects of hundreds to thousands of known and unknown substances and have been applied at regional scales.^28–30^ The spatial variability of these indicators (e.g., decline with distance from sources or global standard deviation) could provide additional information on locally vs globally distributed stressors. Although impacts on humans are not explicitly included in the Planetary Boundary concept, human exposure and health effects at the level of populations and communities may nevertheless provide important evidence of broader biosphere impacts from novel entities in the context of a One Health approach which recognizes the interconnected health of people, animals, and ecosystems, particularly given the comparatively extensive monitoring and data availability for human populations. As a more prospective impact-level indicator, the ‘chemical footprint’ metric could be used to quantitatively connect sources of novel entities to impacts on both ecosystems and human health, while relying on data related to usage and emissions of novel entities as well as on tools translating these into potential impact.^15^

### Interactions with Other Planetary Boundaries

Novel entities act as stressors across planetary boundaries, affecting multiple Earth system processes. Our system of pressure, state, and impact control variables provides a structured way to relate the scale and persistence of anthropogenic stress from novel entities to observed ecosystem impacts and to position that stress within the broader Planetary Boundaries framework. The most explicitly assessed interaction is with the Biosphere Integrity boundary,^6,7,31–33^ where chemical and material proliferation can alter species composition, ecosystem functioning, and biodiversity. For example, widespread exposure to antibiotics and biocides is contributing to antibiotic resistance through the proliferation and global dissemination of resistance genes, affecting biosphere integrity and ecosystem functioning.^34–38^ Novel entities also interact with the climate system and other boundaries.^6^ Chlorofluorocarbons and other ozone-depleting substances drive stratospheric ozone depletion, which is recognized with its own boundary. Many contemporary novel entities have more diffuse effects on multiple boundaries, including greenhouse gas emissions across life cycle stages, formation of radiatively active trace gases or aerosols, and physical effects such as changes in surface properties and albedo.^6,9,39^ They further affect biogeochemical processes by interacting with microbial nutrient cycling and carbon sequestration pathways, often in combination with other anthropogenic stressors.^3,40^ Because of the diversity of novel entities, we must stay vigilant regarding potential unknown or emerging impacts of novel entities at the planetary scale.

## 2 Making Information More Available

### Closing Knowledge Gaps

Data scarcity is a challenge across the entire impact pathway of novel entities, increasingly toward impacts. For many known substances, data at the production stage exist (though are seldom public) but less so regarding where and how they are used, their releases, distribution, accumulation, exposure, and impacts. Data are constrained by analytical limitations and sparse geographical coverage, particularly in low-resourced countries and remote regions with limited infrastructure. Without a comprehensive data set covering the full impact pathway, a comprehensive assessment remains severely constrained. Initiatives such as the planned EU Common Data Platform on Chemicals may provide a starting point for more integrated assessment.^41^ To move ahead, biomonitoring of novel entities in humans could be a useful indicator, noting that production, use, release, and exposure are generally widespread when a novel entity is detectable. Humans are broadly monitored in high-income countries and integrate exposures across uptake pathways, times, and sites. Human biomonitoring, performed under the best ethical practices, can provide evidence of bioaccumulation, long-range transport, and exposure to complex mixtures, including certain regions poorly captured by environmental monitoring. While human health is not an Earth system process and thus is formally outside the purview of the Planetary Boundary framework, accumulation in and impacts on humans can be indicators of exposure-related stress on the biosphere and cases where novel entities have surpassed acceptable levels.

### Toward Comprehensive Monitoring and Data Collection

We propose multiple strategies for improved monitoring, to be adjusted to the subset of novel entities of interest as well as to the emergence of new technologies over time: (i) innovative approaches for inexpensive, standardized, and globally applicable sampling methods and sensors that can generate data even in regions with limited infrastructure,^42–44^ (ii) chemical screening combined with (mixture) effect modeling;^28,29,45^ (iii) effect-based methods including NAMs for mixture effect assessment;^46–48^ (iv) remote-sensing tools (e.g., drone- or satellite-based surveillance with automated image analysis for plastics) where accessibility for sampling is limited;^49^ (v) machine learning approaches to evaluate and cluster chemicals based on their structure and hazard similarities, identify patterns of co-occurrence, and support predictions of distribution, accumulation, exposure, and negative effects (e.g., toxicity, adverse ecological outcomes) where empirical data are lacking.^50–54^ These tools can also help to systematically extract and synthesize relevant information from existing databases (e.g., regulatory data such as registration and authorization dossiers) and to connect fragmented data sets and improve early warning capabilities, among others through impact pathway approaches developed for plastics.^55^ Their effective use requires sustained investment, capacity building, and global coordination since regions facing the highest pollution burdens in many cases have the weakest monitoring capacity and resources for data compilation. Monitoring cannot be a short-term, isolated effort but rather needs to be developed as a global infrastructure that enables continuous, harmonized observation and estimation of pressure, state, and impact of novel entities through strengthened international monitoring networks and long-term institutional support.

## 3 Governance Frameworks and Communication

### Regulatory and Policy-Related Success Stories

Global governance frameworks are essential for addressing widespread pollution. Several international frameworks have successfully led to reductions of releases, distribution, accumulation, and exposure and ultimately led to reduced impacts from novel entities, albeit limited to specific chemicals, e.g., the Stockholm Convention on Persistent Organic Pollutants. The Globally Harmonized System of Classification and Labeling of Chemicals (GHS) defines hazard criteria, setting the basis for many national and regional regulations and policies designed to provide public information on the hazards of chemicals and mixtures. The EU Water Framework Directive integrates chemical and ecological pressures and may be a role model for scaling up. Consistent implementation can stimulate broader international adoption and generate spillover effects to global levels.^56–59^

### Current Obstacles to Effective Governance of Novel Entities

Beyond the scope of existing international governance frameworks, regulation remains fragmented and largely reactive. Even in well-resourced countries, regulatory systems focus narrowly on individual substances, in stark contrast to the more than 350 000 chemicals globally registered for production and use.^60^ With few exceptions, assessments are typically conducted on a substance-by-substance basis and are largely retrospective, often documenting evidence of harm rather than anticipating or preventing it. Even where multilateral environmental agreements exist, they require national implementation, which frequently lags due to limited resources, institutional capacity, or political prioritization.^61^ Beyond fragmented governance, knowledge gaps further constrain effective regulation, e.g., related to confidentiality claims, analytical limitations, and structural barriers to data sharing.^11,62^ Moreover, the complexity of chemical mixtures, multiple interacting stressors, and the vast number of poorly characterized or unknown novel entities substantially complicate conventional risk assessment, let alone regulation, and compliance monitoring.

### Approaches to Overcome these Obstacles

Addressing these structural weaknesses requires a shift toward proactive, systemic governance. The “One Substance One Assessment” approach of the EU, aiming to streamline chemical safety assessments,^63^ could be an important step forward if data are transparent and widely accessible. However, business confidentiality and competition law currently limit data sharing and cooperation among companies.^64^ In contrast, agreements such as the Stockholm Convention and institutions such as the World Health Organization emphasize that restricting access to environmental and human health-related information conflicts with the right to information and public health protection.^65,66^ For substances with insufficient data, a higher level of precaution is warranted. Defining exposure levels associated with no observable or minimal harm may offer an operational basis for governance while explicitly acknowledging uncertainty. Implementing mixture allocation factors,^21,67^ grouping of chemicals with shared hazard characteristics, and promoting chemical simplification strategies could make governance more proactive and protective.^68,69^ Combining concepts such as “essential use” and “safe-and-sustainable-by-design” could provide pathways toward reducing unnecessary chemical complexity and fostering innovation.^70,71^ Extended producer responsibility and full life-cycle cost accounting are also critical. Product prices should reflect environmental and public health costs to incentivize green chemistry and reduce overall chemical intensity. However, these approaches remain insufficiently implemented in a geopolitical context which increasingly sidelines environmental concerns. A way forward might be for coalitions of willing countries to pioneer positive changes in chemicals management. Another avenue might be introducing a new hazard category within the existing GHS framework for substances that are “hazardous to the Novel Entities Planetary Boundary”, as soon as it is defined in an operational way, explicitly linking chemical classification to Earth system stability.

### Public Engagement as a Driver

Public engagement can trigger governance action and market-based change to counteract chemical and material proliferation. Correspondingly, the general public should be a major target audience and cooperation partner for planetary boundaries science, providing civil society with knowledge on potential impacts on ecosystems and human health and empowering them for action. Citizen science activities (e.g., monitoring, plastic cleanup activities) can bring the topic close to people and increase their interest and willingness to support reducing pressures from novel entities. Analytical costs are decreasing, allowing some citizens to commission analyses and act on the basis of the outcomes. Seeking material sufficiency to reduce overconsumption and the related environmental burden cannot, and should not, be placed on individual consumers nor applied uniformly across contexts.^72,73^ In high-income countries, sufficiency may involve consuming less and demanding safer alternatives, while in lower-income settings, the leverage lies in poverty reduction and economic security to reach sufficiency as well as holding international companies and regulatory bodies accountable for safe production and waste disposal practices. Overall, public engagement cannot replace but should support system-level changes.

### Communication about Novel Entities

The term “novel entities“ is not intuitive; hence, we propose to use “chemicals and materials“ as a short name or subtitle for clarification. Effective communication should build on existing knowledge, use accessible language, and highlight solutions to overcome hazardous exposures. Involving social scientists and adopting co-design approaches, where scientists, policy-makers, industry actors, and civil society jointly develop solutions, can help in that respect. Scientists can articulate the co-benefits of action in one domain (e.g., reducing chemical complexity) generating positive outcomes across Planetary Boundaries (e.g., reduced greenhouse gas emissions). Visual communication by artists can make positive future pathways tangible.^74^ Decision-makers need robust evidence on the societal costs of using novel entities, e.g., health care costs, productivity losses, and ecosystem damage.^55,75^ Such information may justify restrictions as economically rational decisions that reduce net societal costs and align with the precautionary and “polluter pays” principles.^76^ It might be useful to remind decision-makers of successful collaboration (e.g., within the European Green Deal)^77^ to revive this spirit. However, decisions ultimately depend on political priorities and are region-specific.

## Final Remarks

Our main proposals include (i) adopting the subtitle “chemicals and materials” to clearly communicate the scope of novel entities; (ii) defining the “safe operating space” as operational precaution under uncertainty rather than absolute safety; (iii) establishing a system of multimetric, complementary pressure – state – impact control variables combined with suitable indicators; (iv) moving engineered biological systems from the Novel Entities to the Biological Integrity boundary while keeping anthropogenically mobilized elements; and (v) strengthening long-term global monitoring, management, and communication regarding novel entities, building upon existing policy, legislation, governance, and involvement of many via citizen science. Together, these measures enhance the operational value of the Novel Entities boundary while maintaining a precautionary approach, consistent with the Planetary Boundaries framework.

## Next Steps

Next steps could include (i) clarifying how existing and emerging concepts and management approaches, such as the envisaged circularity of novel entities can be categorized in the pressures – states – impacts system; (ii) learning from approaches used in other Planetary Boundary domains to translate from the status of the boundary to implementing controls; (iii) exploring to what extent existing data on chemicals and materials can serve to quantify the proposed indicators for the complementary control variables; (iv) examining the needs and design of future data collection which ensures that regulatory reporting and data infrastructures generate the evidence needed to quantify the proposed indicators; (v) developing a system to qualify the level of knowledge available for different chemicals and materials within the broader novel entities framework and guidelines on how to deal with knowledge gaps; (vi) discussing how existing regulations and chemicals management approaches could be extended to cover broader ranges of novel entities and a wider geographical region with respect to monitoring and assessment; (vii) conducting proof-of-concept studies, e.g., under the umbrella of the Organisation for Economic Co-operation and Development (OECD), the Global Framework on Chemicals (GFC), and/or other inclusive international fora, to test the operationalization of the proposed indicators for the complementary control variables; and (viii) supporting existing and emerging multisectoral fora including scientists, regulators, industry, and representatives of civil society to strengthen protective management of chemicals and materials globally. While new ideas and perspectives are needed and welcome, it may also be worthwhile to explore mechanisms that ensure the complementarity of future initiatives in a coordinated manner.
